# Supplementary material for: Current Evidence for Biological Biomarkers and Mechanisms Underlying Acute to Chronic Pain Transition across the Pediatric Age Spectrum
Source: J Clin Med. 2023 Aug 9;12(16):5176. doi: 10.3390/jcm12165176 (PMC10455285; doi:10.3390/jcm12165176)
Supplement: Supplementary file 1 [file jcm-12-05176-s001.zip › jcm-2517340-supplementary.pdf]

Current evidence for biological biomarkers and mechanisms underlying acute to chronic pain transition across the pediatric age spectrum.

I Duff,[1] K Krolick,[2] H Mahmoud and [2*] V Chidambaran[2]

[1]Department of Neurosurgery, Johns Hopkins University, Baltimore, USA
[2]Department of Anesthesia, Cincinnati Children's Hospital, Cincinnati, OH
Undergraduate, Purdue University

* Correspondence: Vidya Chidambaran, MD, MS, FASA; Professor, Department of Anesthesiology, Cincinnati Children's Hospital, Cincinnati, OH 45242, United States of America; Ph. No: 15136368021; Email: vidya.chidambaran@cchmc.org

Supplementary file

Genetics and epigenetics search strategy: When searching the literature an advanced search was conducted in PubMed using the full search-terms listed below for 'pain' AND 'surgery or trauma' AND ('infant' OR 'children and adolescents') AND 'genetics and epigenetics'. Only those infant studies which analyzed the epigenetic changes years after the initial skin-breaks in the NICU were kept in this summary figure (see text for more findings). When searching the literature for 'children and adolescent' findings, only those studies which included a chronic aspect of pain were kept. *FULL SEARCH TERMS- **Pain**: ("pain"[Title/Abstract] OR "nocicepti*"[Title/Abstract] OR "CPSP"[Title/Abstract] OR "PPSP"[Title/Abstract] OR "skin-break"[Title/Abstract]) AND ("surger*"[Title/Abstract] OR "surgical*"[Title/Abstract] OR "trauma*"[Title/Abstract] OR "procedur*"[Title/Abstract] OR "postsurg*"[Title/Abstract] OR "post-surg*"[Title/Abstract] OR "postoperative"[Title/Abstract] OR "post-operative"[Title/Abstract] OR "perioperative"[Title/Abstract] OR "peri-operative"[Title/Abstract]). **Children and Adolescents:** ("toddler*"[Title/Abstract] OR "neonat*"[Title/Abstract] OR "pediatric*"[Title/Abstract] OR "prepubertal" [Title/Abstract]) OR "adolescen*"[Title/Abstract] OR "teenage*"[Title/Abstract] OR "teen*"[Title/Abstract] OR "youth*"[Title/Abstract]). **Infants**: **(**"infant*"[Title/Abstract] OR "newborn*"[Title/Abstract] OR "neonat*"[Title/Abstract] OR "preterm"[Title/Abstract] OR "prenatal*"[Title/Abstract] OR "prematur*"[Title/Abstract] OR "premie*"[Title/Abstract]). **Epigenetics and genetics**: "epigenetic*"[Title/Abstract] OR "epigenom*"[Title/Abstract] OR "microRNA*"[Title/Abstract] OR "miRNA*"[Title/Abstract] OR "histone-acetylation"[Title/Abstract] OR "histone-methylation"[Title/Abstract] OR "DNA-methylation"[Title/Abstract] OR "5'-C-phosphate-G-3'"[Title/Abstract] OR "CpG"[Title/Abstract] OR "CpGs"[Title/Abstract] OR "CGI"[Title/Abstract] OR "CGIs"[Title/Abstract] OR "chromatin"[Title/Abstract] OR "CpG-island-methylator-phenotype"[Title/Abstract] OR "CIMP*"[Title/Abstract] OR "methyltransferase*"[Title/Abstract] OR "meQTL*"[Title/Abstract] OR "methylation-quantitative-trait-loci"[Title/Abstract] OR "chromatin-accessibility"[Title/Abstract] OR "chromatin-modification*"[Title/Abstract] OR "GWAS"[Title/Abstract] OR "genome-wide-association-stud*"[Title/Abstract] OR "genetic*"[Title/Abstract] OR "genomic*"[Title/Abstract] OR "gene"[Title/Abstract] OR "transcriptome*"[Title/Abstract] OR "transcription*"[Title/Abstract] OR "mRNA"[Title/Abstract] OR "RNA"[Title/Abstract] OR "DNA"[Title/Abstract] OR "SNP*"[Title/Abstract] OR "single-nucleotide-polymorphism*"[Title/Abstract] OR "eQTL"[Title/Abstract] OR "expression-*

quantitative-trait-loci"[Title/Abstract] OR "polygenic-risk-score"[Title/Abstract] OR "methylation-risk-score"[Title/Abstract].
